# Intensive removal of signal crayfish (*Pacifastacus leniusculus*) from rivers increases numbers and taxon richness of macroinvertebrate species

**DOI:** 10.1002/ece3.903

**Published:** 2014-01-23

**Authors:** Tom P Moorhouse, Alison E Poole, Laura C Evans, David C Bradley, David W Macdonald

**Affiliations:** 1 Wild CRU, Zoology, University of Oxford, The Recanati-Kaplan CentreTubney House, Abingdon Road, Tubney, Abingdon, OX13 5QL, U.K; 2 APEM Limited, Riverview A17 Embankment Business ParkHeaton Mersey, Stockport, SK4 3GN, U.K

**Keywords:** American signal crayfish, aquatic macroinvertebrate numbers, density, macroinvertebrate taxon richness, manual removal by trapping, *Pacifastacus leniusculus*

## Abstract

Invasive species are a major cause of species extinction in freshwater ecosystems, and crayfish species are particularly pervasive. The invasive American signal crayfish *Pacifastacus leniusculus* has impacts over a range of trophic levels, but particularly on benthic aquatic macroinvertebrates. Our study examined the effect on the macroinvertebrate community of removal trapping of signal crayfish from UK rivers. Crayfish were intensively trapped and removed from two tributaries of the River Thames to test the hypothesis that lowering signal crayfish densities would result in increases in macroinvertebrate numbers and taxon richness. We removed 6181 crayfish over four sessions, resulting in crayfish densities that decreased toward the center of the removal sections. Conversely in control sections (where crayfish were trapped and returned), crayfish density increased toward the center of the section. Macroinvertebrate numbers and taxon richness were inversely correlated with crayfish densities. Multivariate analysis of the abundance of each taxon yielded similar results and indicated that crayfish removals had positive impacts on macroinvertebrate numbers and taxon richness but did not alter the composition of the wider macroinvertebrate community. *Synthesis and applications*: Our results demonstrate that non-eradication-oriented crayfish removal programmes may lead to increases in the total number of macroinvertebrates living in the benthos. This represents the first evidence that removing signal crayfish from riparian systems, at intensities feasible during control attempts or commercial crayfishing, may be beneficial for a range of sympatric aquatic macroinvertebrates.

## Introduction

Globally, ecosystems are highly susceptible to biological invasions (Parker et al. 1999), and invasive species are a major a driving force of extinctions (Lowe et al. 2000). Invasions may have detrimental effects on the biodiversity (Zavaleta et al. 2001) and genetic diversity (e.g., Fitzpatrick et al. 2010) of native species and alter the food web structure of ecosystems (Taylor et al. 1984). Clavero and Garcia-Berthou (2005) analyzed 680 animal extinctions reported in the IUCN Red List database: of 170 cases with known causes, 54% (91) resulted at least partially from the impacts of alien species.

Freshwater habitats are especially at risk from alien species (Heywood 1995), and invasions are the principal source of biodiversity loss in such ecosystems (Vitousek et al. 1996; Sala et al. 2000; Hooper et al. 2005). Crayfish are a particularly pervasive freshwater invasive: worldwide nearly 30 species of crayfish have exploited aquatic habitats outside their native area, due to human activity (Gherardi 2010). The American signal crayfish (*Pacifastacus leniusculus* Dana), originally from western North America, are invasive in 21 countries (Lewis 2002) and in the UK are rapidly replacing the native white-clawed crayfish *(Austropotamobius pallipes* Lereboullet) (Crawford et al. 2006).

Invasive crayfish have negative effects upon aquatic macrophytes (Creed 1994; Lodge et al. 1994), amphibians (Axelsson et al. 1997), fish (Guan and Wiles 1998), and benthic aquatic macroinvertebrates (Guan and Wiles 1998; Nystrom et al. 1999; McCarthy et al. 2006). Invasive crayfish may reduce the abundance of snails (Hanson et al. 1990; Nystrom et al. 1999; McCarthy et al. 2006), dipterans (McCarthy et al. 2006), and chironomids, Trichoptera, Ephemeroptera, and Coleoptera (Guan and Wiles 1998), although in the latter case effects vary with crayfish age, macroinvertebrate species, and season. Crayfish also affect other macroinvertebrates indirectly through reduction of food sources (Nystrom 2002). These effects, coupled with high consumption rates and rapid population growth (Nystrom 2002), mean that crayfish pose some of the greatest threats to freshwater biodiversity worldwide (e.g., Clavero and Garcia-Berthou 2005; Macdonald et al. 2006).

Mechanical, biological, and chemical control methods have been used in attempts to eradicate signal crayfish, but this has never been achieved (e.g., Gherardi et al. 2011). Eradication may not, however, be necessary to control an invasive species and restore ecosystem function (Simberloff 2009). Few studies for any taxonomic groups demonstrate the feasibility and benefits of invasive species control (Simberloff 2009), but some recent research on invasive crayfish has focussed on optimizing the effectiveness of noneradication control strategies (e.g., Rogowski et al. 2013) and on assessing the effects of those strategies on impacted biota, in particular upon the structure and abundance of macrophyte and macroinvertebrate communities (Usio et al. 2009; Hansen et al. 2013). These latter studies took place in experimental enclosures in marshland habitats (Usio et al. 2009), or in lakes in which the densities of the crayfish have naturally fluctuated (Kreps et al. 2012) or were experimentally, and substantially, reduced over periods of years (Hansen et al. 2013).

In this study, we investigate the value of short-term, intensive American signal crayfish control strategies for mitigating their impacts on the benthic macroinvertebrate fauna in river habitats. We report the results of replicated experimental removals of *P. leniusculus* from two tributaries of the River Thames, UK, in lowland agricultural catchments. The removals were designed to mimic removal rates that would be feasible in a large-scale control attempt and which are typical of commercial crayfishing enterprises. We test the hypothesis that in reaches where the signal crayfish are removed macroinvertebrate numbers and taxon richness will increase compared with control reaches.

## Material and Methods

### Study area and experimental design

The study was conducted on two 1-km stretches of river, each containing six “sections.” Each river contained two “experimental sections”: a removal section (the treatment) and a nonremoval section (the experimental control), both 100 m in length and separated by buffer of a minimum of 500 m in length in which no crayfish trapping took place. Each experimental section was bounded by two 90-m “flanking” sections (up and downstream) where crayfish were not trapped but in which macroinvertebrates were sampled. In total, the study therefore comprised two rivers, each containing two experimental sections (one removal and one nonremoval), each bounded by two flanking sections. The two rivers were the Evenlode and Thame, both located in Oxfordshire, UK (UK national grid references were as follows: Evenlode SP 437 112; 439 117 and Thame: SP 672 069; 677 066).

Protocol for crayfish trapping is described in detail elsewhere (Moorhouse and Macdonald 2011a,b2011b), but in brief, trapping was carried out in experimental removal and nonremoval sections simultaneously with four sessions, each of nine consecutive days, over a 4-month period, beginning in May 2010. At each river, trapping sessions were separated by a 3-week period. The cylindrical crayfish traps used were 50 cm in length and 20 cm in diameter and commercially produced (Trappy™ crayfish traps; Trappy, Virserum, Sweden). Crayfish traps were baited with sardines and laid in pairs, one either side of the river, every 5 m along the length of each experimental section, resulting in 21 pairs of traps running the length of each experimental section. In the removal sections, half of the trapped crayfish were marked and returned – for a separate study on crayfish movement distances and growth rates (Moorhouse and Macdonald 2011a,b2011b) – while the other half were removed and humanely destroyed by freezing (RSPCA 2003). In nonremoval sections, all crayfish captured were marked and returned (for further details, see Moorhouse and Macdonald 2011a).

Six sampling kits for aquatic macroinvertebrates were placed in each of the experimental sections at a spacing of one kit every three or four crayfish traps (between traps 1/2, 5/6, 9/10, 12/13, 16/17, and 20/21). Kits were also placed in the flanking sections at distances of 30 m, 60 m, and 90 m from the ends of the experimental sections. Each river therefore contained a total of 24 sampling kits: six in each experimental section and six in each pair of flanking sections. Sampling kits comprised a pair of standard colonization units (hereafter referred to as “samplers”) fixed together. The samplers were 150 × 100 mm, Standard Aufwuchs Unit Samplers, based upon the design of Girton and Hawkes (DEN/WC 1984), and composed of white polypropylene pall rings and 1 mm white polyester netting, purchased from EFE GB Nets (http://www.efe-gbnets.com). One sampler was left uncovered (hereafter an “open” sampler), while the other was modified by enclosing it in a plastic mesh cage (hereafter a “closed” sampler) to prevent larger crayfish from preying on collected macroinvertebrates. The mesh size was 5 mm, but some small (<10 mm diameter) macroinvertebrates and crayfish were able to enter through the edges of the boxes. Each sampling kit was weighed down with gravel bags and fixed to the riverbank by a rope. The exact location of each kit was recorded using a Garmin eTrex GPS. Each sampling kit was emptied every 4 weeks, three times between June and September 2010, the first occasion being 4 weeks after the initial capture session of crayfish and immediately prior to the next crayfish capture session (so at the beginning of sessions 2, 3, and 4).

Captured macroinvertebrates were preserved in 90% ethanol solution for a maximum of 6 months prior to identification and counting.

The two rivers were selected to ensure low environmental variability between rivers and sections. River features included smooth flow type, silt as the main substrate, emergent broadleaf, emergent reeds, and amphibious vegetation as the predominant cover. A River Habitat Survey (Raven et al. 1998) was carried out at each river for both nonremoval and removal sections which confirmed these environmental similarities.

### Sample analysis

Samples were analyzed in APEM Ltd's laboratories (Heaton Mersey, Stockport, U.K.) to a UKAS-accredited procedure, which is compatible with standard Environment Agency (a British statutory body) procedures. Samples were washed within the confines of a fume cupboard using a 500-*μ*m sieve to separate preservative and fine silt from the retained sample fraction. Samples were sorted, with up to three good quality specimens of each taxon put into a vial containing 70% IMS solution to facilitate quality assurance. All the remaining animals were removed and placed in a separate vial. All sample material was transferred to 70% industrial methylated spirits solution as a long-term preservative.

Macroinvertebrates were identified to species level where possible for all groups except Sphaeriidae, Oligochaeta, Hydracarina, Simuliidae, and Chironomidae, which were not identified further; other Diptera were identified to family or genus level. The numbers of individuals of each taxonomic group in each sample were counted.

### Statistical analysis

#### Did removals affect crayfish densities in the experimental sections?

We constructed general linear models, in Minitab, of the factors affecting mean catch per unit effort (CPUE) of crayfish in the removal and nonremoval experimental sections. Mean CPUE was analyzed only for the first day of trapping for removal sessions 2–4, because this measures the distribution of crayfish prior to any removals in that session and after any between-sessions immigration and therefore gives an indication of the maximum density supported by the stretch during the intersession period (see Moorhouse and Macdonald 2011b). Available explanatory variables to explain variations in CPUE were treatment, river, session (entered as a covariate, to test for trends in CPUE over time), and distance from the upstream and downstream edges of the removal section (0–50 m, where the pair of traps at 50 m were in the center of the removal section, and pairs at 0 m were at either end). This latter variable was included because immigration into removal sections over the 3 weeks between sessions may be expected to create a gradient of crayfish densities to which the macroinvertebrate numbers and taxon richness might respond (e.g., Moorhouse and Macdonald 2011c).

#### Did removals affect macroinvertebrate counts and taxon richness in the experimental sections?

We conducted separate analyses for open samplers (those without a protective mesh cage) and closed samplers (with mesh cages, which were assumed to be less affected by crayfish predation) to test for the effect of our experimental manipulations on the macroinvertebrate community. The results from these samplers were treated separately because the mesh may have influenced both the rate of colonization of the samplers and the varieties of macroinvertebrates they supported, meaning that the types of sampler were not directly comparable (see Discussion). In these analyses, macroinvertebrate numbers and taxon richness were the responses in separate repeated-measures models, and treatment, river, session (entered as a factor because any temporal trend would be accommodated in mean CPUE, below), and the mean number of crayfish captured in the closest two pairs of crayfish traps (“mean CPUE”, a measure of crayfish density immediately surrounding the sampler) were entered as explanatory variables. The analyses were conducted in Program R (R Core Team 2013), using the lme function, with sampler specified as a random factor. Taxon richness and macroinvertebrate count were square-root transformed to meet the assumptions of the test.

#### Did removals affect macroinvertebrate counts and taxon richness in the flanking sections?

We anticipated that our experimental manipulation would result in lowered densities the flanking sections around removal, but not nonremoval, experimental sections due to crayfish migration down a density gradient (Moorhouse and Macdonald 2011c) and that the manipulation may therefore lead to differences between flanking sections in macroinvertebrate counts and taxon richness. CPUE was not measured directly in these sections, and so, analyses were constructed using river, session, and treatment (whether the flanking sections bordered a removal or nonremoval experimental section) as explanatory variables. The analyses were conducted in R, using the lme function, with sampler specified as a random factor. Taxon richness and macroinvertebrate count were square-root transformed to meet the assumptions of the test.

#### Did crayfish removals affect macroinvertebrate species composition?

We used the manyglm function of R, within the mvabund package, to assess how our experimental manipulation affected abundances of individual taxa in the macroinvertebrate community (Wang et al. 2012). Manyglm fits a separate, univariate, generalized linear model to the recorded abundance of each taxon – in this case counts of the taxon on each sampler – and relates each abundance to a common set of explanatory variables to create a multivariate analysis across taxa. The function uses resampling-based hypothesis testing to make community-level and taxon-specific inferences, returning a multivariate analysis testing which factors or environmental variables are associated with the multivariate abundances (i.e., with the community of species as a whole) (Wang et al. 2012; and see Gibb and Cunningham 2013 and Holmstrup et al. 2013 for recent usage). We ran the manyglm model for experimental (removal and nonremoval) sections; the response variable was the count of each taxon captured on each sampler, and the explanatory variables were mean CPUE, river, and treatment. We performed separate analyses for each session to prevent pseudoreplication from repeated measures on each sampler.

## Results

### Did the removals affect crayfish densities in the experimental sections?

A total of 6181 crayfish was removed from the two 100-m removal stretches over the course of the study. The impacts of removals on CPUE of crayfish within and between capture sessions are detailed in Moorhouse and Macdonald (2011b), but here, we present a new analysis of these data, focussing on how between-sessions densities varied spatially.

**Figure 1:**
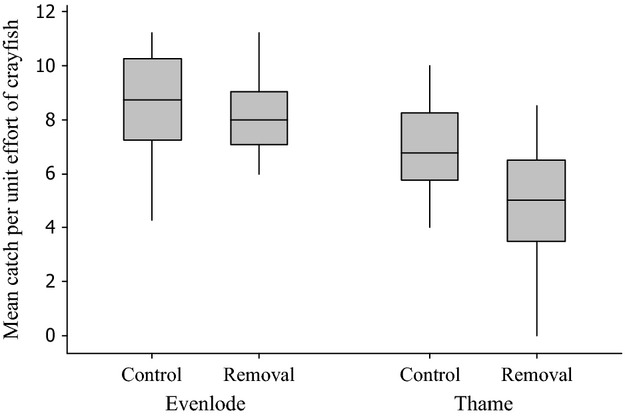
Boxplot showing the effect of crayfish removals on mean catch per unit effort (CPUE) on the rivers Evenlode and Thame. Boxes represent the median and interquartile range. Whiskers represent extreme values.

CPUE for the first day of each of sessions 2–4 was affected by an interaction between treatment and distance from the edge of the section, such that CPUE decreased with increasing distance in removal sections and increased with increasing distance in nonremoval sections (effect of distance × treatment on CPUE, *F*_1,126_ = 12.65, *P* < 0.001; Table 1). Marginal mean CPUE for traps at the edge of removal sections (distance = 0 m) was 7.2 crayfish, whereas for traps in the center of removal sections (distance = 50 m), this figure was 5.4. For nonremoval sections, these figures were 7.6 and 9.5 crayfish per trap, respectively. CPUE also differed between rivers, such that mean CPUE at the Thame and Evenlode sites was 4.8 and 7.8, respectively (Fig. 1). There was no evidence, however, that the interaction between distance and treatment differed between rivers (effect of distance × treatment × river, *F*_1,122_, *P* > 0.3 from a model in which this interaction was included). There was also no evidence that mean crayfish densities in the removal stretches decreased over concurrent sessions (effect of treatment × session *F*_1,125_ = 0.21, *P* = 0.647) from a model in which this interaction was included.

**Table 1:** Factors affecting catch per unit effort of crayfish on the first day of trapping in sessions 2–4 in the central sections of each site.

Source	Numerator and denominator df	*F*	*P*
River	1, 126	44.74	<0.001
Treatment	1, 126	0.29	0.594
Session	1, 126	1.91	0.169
Distance	1, 126	0.13	0.724
Distance × Treatment	1, 126	12.65	0.001

### Did removals affect macroinvertebrate counts and taxon richness in the experimental sections?

**Figure 2:**
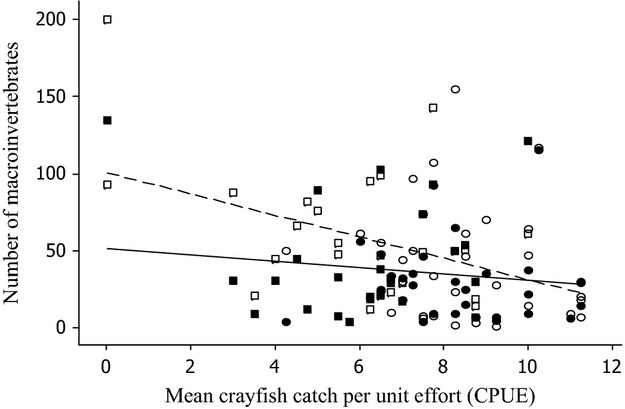
The effect of variations in the densities of crayfish, as measured by CPUE, on the number of individual macroinvertebrates collected on open (open symbols) and closed (shaded symbols) samplers on the rivers Evenlode (circles) and Thame (squares). Regression lines are for demonstration purposes and include only the effects of CPUE on invertebrate numbers for open (dotted line) and closed (solid line) samplers.

**Figure 3:**
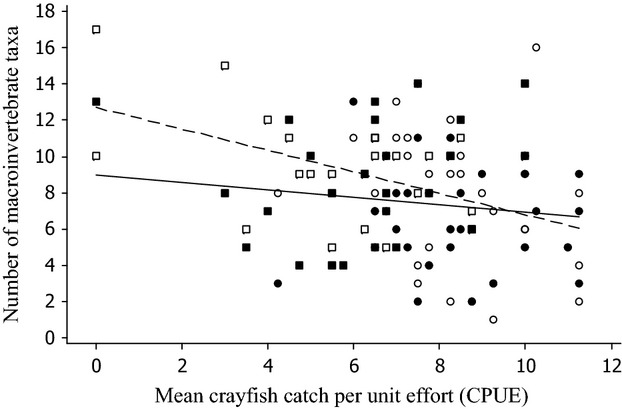
The effect of variations in the densities of crayfish, as measured by CPUE, on the number of macroinvertebrate taxa collected on open (open symbols) and closed (shaded symbols) samplers on the rivers Evenlode (circles) and Thame (squares). Regression lines are for demonstration purposes and include only the effects of CPUE on invertebrate numbers for open (dotted line) and closed (solid line) samplers.

The relationship of macroinvertebrate counts and richness with mean CPUE differed between open and closed samplers (Figs 3). Macroinvertebrate counts from open samplers were negatively correlated with crayfish densities (effect of CPUE *F*_1,30_ = 14.62, *P* < 0.001; Table 2a), such that across the range of mean CPUE used in the analysis (mean CPUE range 0.0–11.3), marginal mean macroinvertebrate counts varied from 110.4 to 18.5 (Fig. 2). Similarly, macroinvertebrate taxon richness for open samplers was negatively correlated with mean CPUE (effect of mean CPUE *F*_1,30_ = 12.76, *P* = 0.0012; Table 2b), such that taxon richness was 13.5 when mean CPUE was 0.0 and 5.6 when mean CPUE was 11.3 (Fig. 3). In both analyses, treatment (which was included to account for any external differences between removal and nonremoval stretches) and mean CPUE were confounded, and models in which treatment was removed gave slightly higher effects of mean CPUE (*F*_1,30_ = 15.51, *P* < 0.001, and *F*_1,30_ = 13.13, *P* = 0.0011 for counts and richness, respectively).

**Table 2:** Factors affecting (a) total macroinvertebrate counts, (b) taxon richness for “open” samplers (those without a mesh cage), (c) total macroinvertebrate counts, and (d) taxon richness for “closed” samplers (those with a mesh cage) in the experimental sections.

Source	Numerator and denominator df	*F*	*P*
(a)			
River	1, 21	2.76	0.111
Treatment	1, 21	0.735	0.401
Session	2, 30	2.20	0.129
Mean CPUE	1, 30	14.62	0.0006
(b)			
River	1, 21	5.98	0.0233
Treatment	1, 21	0.13	0.724
Session	2, 30	6.41	0.0048
Mean CPUE	1, 30	12.76	0.0012
(c)			
River	1, 21	0.94	0.344
Treatment	1, 21	0.0033	0.954
Session	2, 28	2.67	0.086
Mean CPUE	1, 28	1.18	0.287
(d)			
River	1, 21	5.41	0.030
Treatment	1, 21	1.86	0.187
Session	2, 28	2.53	0.098
Mean CPUE	1, 28	0.007	0.931

For closed samplers, neither macroinvertebrate counts nor taxon richness was significantly affected by mean CPUE (effect of mean CPUE *F*_1,28_ = 1.18, *P* = 0.287, and *F*_1,28_ = 0. 28, *P* = 0.931 for counts and richness, respectively; Table 2c,d; Figs 3). In both cases, removal of treatment from the model did not substantively improve the association between mean CPUE and the response (effect of mean CPUE *F*_1,28_ = 0.99, *P* = 0.389, *F*_1,28_ = 0.20 *P* = 0.662, for counts and richness, respectively).

We repeated these analyses for a subset of data limited to values of CPUE > 3, to exclude the possibility that extremely low levels of CPUE, potentially resulting from factors other than removals, influenced the relationship between CPUE and invertebrate counts and taxon richness. The results of this sensitivity test did not differ from those stated above.

The difference in mean macroinvertebrate counts between open samplers (50.0; SE 4.7) and closed samplers (36.7; SE 4.8) was marginally nonsignificant (effect of cage F_1,44_ = 4.01, *P* = 0.051) in an analysis that also included effects of river, treatment, and session. Taxon richness did not differ between samplers (effect of cage *F*_1,44_ = 2.44, *P* = 0.125).

### Did removals affect macroinvertebrate counts and taxon richness in the flanking sections?

Macroinvertebrate counts in the open samplers in flanking sections were affected by treatment, such that mean counts in the sections flanking removals were higher (35.2 as opposed to 25.6) than in sections flanking nonremovals (effect of treatment *F*_1,21_ = 4.57, *P* = 0.045; Table 3a). Taxon richness, however, did not vary with treatment (effect of treatment *F*_1,21_ = 1.38, *P* = 0.253; Table 3b). For the closed samplers, neither macroinvertebrate counts nor taxon richness varied with treatment (Table 3c,d). There was no evidence in any analysis for a significant effect of an interaction between distance (samplers at 30, 60, or 90 m from the edge of the experimental sections, entered as a covariate) and treatment (effect of distance × treatment *P* > 0.6 in all cases), and this term was therefore excluded from the final models.

**Table 3:** Factors affecting (a) total macroinvertebrate counts, (b) taxon richness for “open” samplers (those without a mesh cage), (c) total macroinvertebrate counts, and (d) taxon richness for “closed” samplers in the flanking sections.

Source	Numerator and denominator df	*F*	*P*
(a)			
River	1, 21	7.09	0.0146
Treatment	1, 21	4.57	0.0445
Session	3, 69	1.89	0.139
(b)			
River	1, 21	7.87	0.011
Treatment	1, 21	1.38	0.253
Session	3, 69	2.33	0.082
(c)			
River	1, 21	2.87	0.105
Treatment	1, 21	1.60	0.220
Session	3, 69	3.25	0.027
(d)			
River	1, 21	1.71	0.21
Treatment	1, 21	0.06	0.81
Session	3, 69	3.62	0.017

### Did crayfish removals affect macroinvertebrate taxon composition?

**Figure 4:**
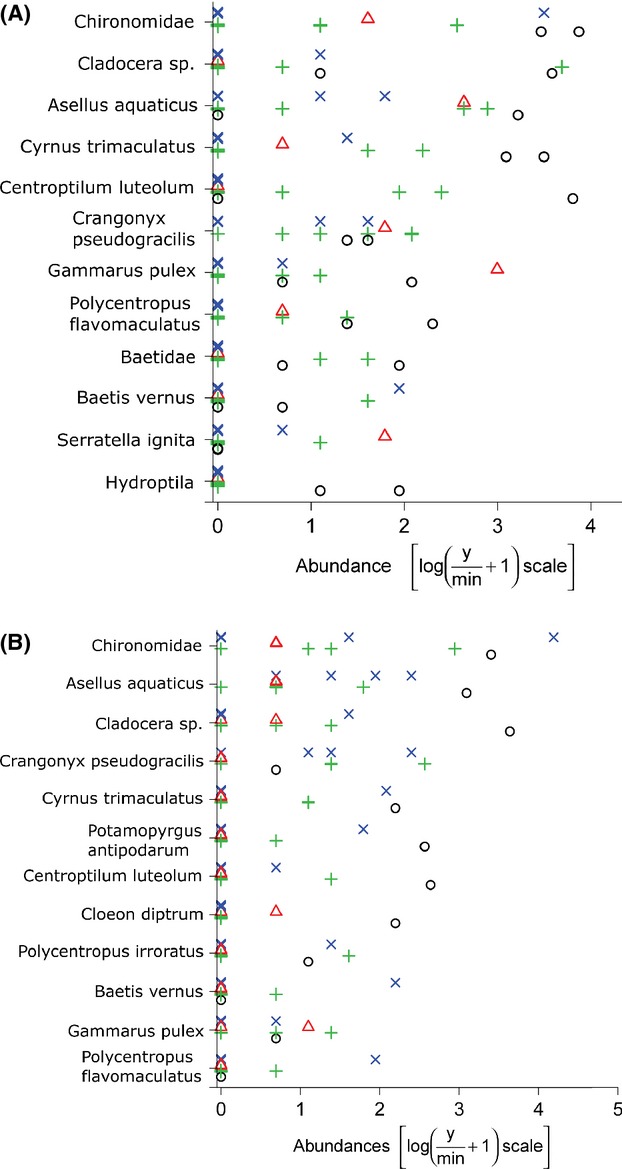
The effect of CPUE on the abundance of the 10 most abundant species of noncrayfish macroinvertebrate for A) open samplers and B) closed samplers in session 2. Data are presented only for this session for demonstration purposes, but are representative of the relationship between species abundance and CPUE in session 4. For presentation purposes, CPUE was divided into four categories, denoted as circles (CPUE 0–2.9), triangles (CPUE 3–5.9), pluses (CPUE 6–8.9), and crosses (CPUE 9–11.9).

The mvabund analysis of the count of individuals of each taxon on each sampler provides a degree of evidence that crayfish densities affected the abundance of macroinvertebrate taxa occupying open, but not closed, samplers in the experimental sections (Table 4a,b). For open samplers, CPUE was associated with variations in species abundance for session 2 (Dev. = 68.05, *P* = 0.046, Table 4a; Fig. 4A) and session 4 (Dev. = 74.8, *P* = 0.033; Table 4a), but not for session 3 (Dev. = 37.9, *P* > 0.34; Table 4a). For closed samplers, there was no evidence of a change in species abundance with different levels of CPUE (Dev. < 52.4, *P* > 0.11 in all cases; Table 4b; Fig. 4B). In all analyses, river had a significant effect on the abundances of macroinvertebrate taxa (Table 4a,b).

**Table 4:** Factors affecting the species distribution of macroinvertebrates across (a) “open” samplers (those without a mesh cage) and (b) “closed” samplers in the experimental sections.

	Session 2			Session 3			Session 4		
Source	Resid. df and df diff.	Dev.	*P*	Resid. df and df diff.	Dev.	*P*	Resid. df and df diff.	Dev.	*P*
(a)									
River	11,1	106.78	0.004	18,1	77.98	0.006	22,1	93.73	0.006
Treatment	10,1	52.88	0.104	17,1	45.42	0.149	21,1	43.88	0.314
Mean CPUE	9,1	68.05	0.046	16,1	37.93	0.343	20,1	74.84	0.033
(b)									
River	9,1	65.01	0.031	18,1	61.19	0.025	22,1	69.72	0.019
Treatment	8,1	40.09	0.135	17,1	34.83	0.321	21,1	38.29	0.384
Mean CPUE	7,1	52.4	0.116	16,1	36.48	0.439	20,1	49.61	0.167

Univariate analyses for each individual taxon demonstrated no relationship between CPUE and abundance, either for open or closed samplers (Dev. < 12.55 and *P* > 0.093 in all cases). Univariate analyses of the effect of river uncovered significant effects only for two taxa: Chironomidae (Dev. = 11.3, *P* = 0.021, mean count 0.75 and 24.8 for Evenlode and Thame, respectively) and Baetidae (Dev. 11.95, 0.018, mean count 0.0 and 2.6, respectively) – and only among open samplers in session 2.

Differences in taxon abundance between open and closed samplers were found in sessions 3 and 4 (multivariate analysis, Dev. = 57.74, *P* = 0.035; Dev. = 126.55, *P* = 0.001, respectively), but not in session 2 (Dev. = 34.57, *P* > 0.50), in a separate multivariate mvabund analysis in which river and treatment were also included. Univariate analyses demonstrated no relationship between sampler type and the abundance of individual taxa (Dev. < 8.58, *P* > 0.16 in all cases).

## Discussion

In the experimental sections, both the numbers and taxon richness of macroinvertebrates were inversely correlated with mean CPUE of crayfish in open but not closed samplers. The effect of variations in mean CPUE on the counts from open samplers was substantial: The range of mean macroinvertebrate counts was 19–110 across the range of CPUE measured in this study, representing a fivefold increase in macroinvertebrate numbers at the lowest crayfish densities. Similarly, taxon richness ranged from 6 to 14, meaning that the number of taxa represented was almost tripled at the lowest crayfish densities. These findings are consistent with expectations if predation pressure from signal crayfish were a principal determinant of presence and abundance for a range of macroinvertebrate species, and the reduction in densities (as measured by CPUE) from our removals diminished this predation pressure sufficiently to permit an increase in numbers and types of macroinvertebrates on the samplers.

The above interpretation requires that the principal determinant of variations in mean CPUE was the removal of crayfish, which is likely to be the case. Moorhouse and Macdonald (2011b) demonstrate that crayfish removals at these sites had significant impacts on CPUE both within and between capture sessions. Our present analysis reveals that measured CPUE varied between treatments, and with the distance of a given crayfish trap from the edge of an experimental section: densities of crayfish were lowest (5.4 per trap) in the center of the 100-m removal sections and higher at the upstream and downstream margins (7.2 per trap). Conversely, in the nonremoval sections, CPUE was highest at the center of the section (9.5 per trap) and lowest at the margins (7.5 per trap). The pattern of CPUE in the removal sections (measured on the first capture day of sessions 2–4) could occur if decreases in crayfish densities during capture sessions were partially compensated, during the 3-week interim periods, by crayfish immigrating from outside of the section (e.g., Moorhouse and Macdonald 2011c). It is less clear what may have caused the reverse pattern in the nonremoval sections, but it could have arisen from the bait used in the trapping study attracting crayfish to the center of the trapped stretch. Regardless of the mechanism, however, the distribution of crayfish densities varied between removal and nonremoval sections, and the most plausible explanation for this is the removal of 6181 crayfish over four capture sessions.

The lack of significant association between CPUE and either counts or taxon richness for the closed samplers plausibly derives from two effects of covering the samplers with mesh netting. Firstly, the mesh is likely to lower the rate of predation by preventing access to the sampler for relatively large signal crayfish, those which typically move further and are more aggressive (see Moorhouse and Macdonald 2011a,b2011b) and which are therefore the most likely to find, and to compete successfully for, resources (e.g., Ranta and Lindstrom 1992, 1993; Barki et al. 2006; Herberholz et al. 2007). Secondly, the mesh cages – by acting as a partial barrier – may reduce the rate at which samplers are colonized by macroinvertebrates. This possibility is supported by the lower mean macroinvertebrate counts (37 as opposed to 50, a difference that was, however, nonsignificant) on closed samplers, and the evidence, albeit only from sessions 3 and 4, from the mvabund analysis that the abundance of taxa on closed samplers was lower than on open samplers. These observations constitute a degree of evidence that the cages limited the numbers of macroinvertebrates that closed samplers could accumulate and in so doing may have reduce the size of any potential treatment differences between removal and nonremoval sections.

It was also possible that the mesh cages may also have preferentially excluded larger-bodied macroinvertebrate species from the closed samplers. However, no statistically significant effect of sampler type was discerned on taxon richness, and the lack of evidence for any effect of cage on a given taxon in the univariate mvabund analyses suggests that while the addition of cages may have lowered the overall abundance of macroinvertebrates, this did not affect any taxon more than the others. Nevertheless, data from closed and open samplers were employed in separate analyses due to the likelihood of the samplers accumulating their fauna at a different rate and therefore forming experimental substrates that were not strictly comparable within the same analysis.

In the flanking sections, where no crayfish trapping occurred, macroinvertebrate counts, but not taxon richness, varied with treatment for the open samplers. For closed samplers, there was no discernible effect of treatment on either measure. These results suggest that immigration into removal, but not nonremoval, sections lowered densities in the flanking sections sufficiently to reduce crayfish predation rates on the samplers. Mean taxon richness in the open samplers in the flanks was 7.8 and 6.9 for removal and nonremoval treatments, respectively. While this difference was nonsignificant, the direction of the difference was consistent with results from the experimental sections, possibly suggesting that the same mechanism was applying, but reduced in intensity. The lack of effect of the distance of the sampler (30, 60 or 90 m) from the experimental section may imply that emigration occurred over distances longer than the 90-m flanking sections; this would accord with the findings of Moorhouse and Macdonald (2011c) who found that, due to immigration, the total population from which crayfish removals are drawn will extend at least 200 m upstream and downstream of the trapped section in riparian habitats.

The mvabund multivariate analyses revealed that across the macroinvertebrate species, decreasing CPUE was associated with increasing abundance on open, but not closed samplers in sessions 2 and 4. No such association was present in session 3. These findings partially corroborate the conclusion that the removals resulted in increased abundances of macroinvertebrates. The lack of univariate effects of CPUE on any given taxon implies that crayfish removals did not affect the abundance of any taxon more than another and that the species composition on the samplers remained approximately the same: Abundances of all taxa responded in a similar fashion to the experimental treatments.

The abundance of macroinvertebrates on the samplers varied between rivers in all mvabund analyses (Table 4). The two study rivers were selected to ensure a high degree of similarity in physical characteristics; particularly, bank structure and bankside vegetation varied little between sites according to our RHS survey. However, other factors, such as variations in water temperature and flow velocity (Extence et al. 1999) or fine sediment (Wood and Armitage 1997), may still have resulted in differences in the distribution of abundances of macroinvertebrate taxa or the rates at which they colonized the samplers. Any such differences, however, do not affect the conclusion that both the number of macroinvertebrates and the taxon richness inversely varied with CPUE on each river.

Previous studies of the effects of crayfish control or reductions in their densities have recorded mixed effects on the macroinvertebrate community. Usio et al. (2009), working with experimental enclosures in marsh habitats, concluded that the per-capita impacts of signal crayfish on communities increase dramatically as individual crayfish become larger, and so control by manual removal, which has a well-reported bias toward the removal of the largest individuals (e.g., Abrahamsson 1966; Guan and Wiles 1996; Westman et al. 1999) may be effective at mitigating their worst impacts. Hansen et al. (2013), following an 8-year removal of invasive rusty crayfish (*Orconectes rusticus*) from a closed system (a 64 ha lake), recorded increases in the abundance of native crayfish and fish species, as well as of macrophytes in some habitats. The macroinvertebrate response, however, varied among families and habitats: Gastropod density increased by 300-fold in cobble habitat, while densities of Ephemeroptera, Odonata, and Amphipoda, which may have been indirectly facilitated by rusty crayfish, declined in certain habitats. In a separate study, Kreps et al. (2012) recorded large reductions in snail abundance in two lakes, in which populations of rusty crayfish increased, but no corresponding increase in snail abundance in two further lakes, in which rusty crayfish abundance subsequently declined.

Our study differs from those listed above in taking place in an open riparian system, a feature of which is continual movement of taxa through the study area, and which does not have the diversity of water depths and habitats recorded in the studies of Hansen et al. (2013) and Kreps et al. (2012). Similarly, our study area lacked any native crayfish, which were present in Hansen et al. (2013) study site (the virile crayfish, *Orconectes virilise*) and which may themselves impact upon macroinvertebrate community when released from competition with an invasive competitor. We also recorded general increases in macroinvertebrate counts and taxon richness over relatively short time periods (4 months) and by relying entirely upon colonization sampling at the benthos. Our results are therefore representative of only short-term trends and of that proportion of the macroinvertebrate community that is amenable to such sampling. Colonization samplers of various types have previously been shown to provide representative samples when compared to other methods (e.g., Boothroyd and Dickie 1989; Whitehurst 1991; Depauw et al. 1994; Czerniawska-Kusza 2004 – but see Turner and Trexler 1997; Blocksom and Flotemersch 2005 who argue that several complementary methods are required for a complete assessment of the benthic community), and the time period over which they were employed in the present study has been shown to produce stable results (Boothroyd and Dickie 1989). While it remains possible that our approach may have excluded some species from analysis, our use of these samplers is unlikely to have materially altered the outcome of our study.

Our results demonstrate that while removal programmes cannot hope to eradicate populations of signal crayfish (e.g., Holdich et al. 1999; Peay 2001), if they are sufficiently intensive, they may quickly result in local increases in the total number of individual macroinvertebrates, and the number of taxa they represent. Our study took place over a 4-month period, and so we were unable to examine long-term impacts of the removals on the macroinvertebrate population. However, the fact that some positive impacts of the removal were discernible in the flanking sections where no removals occurred provides some hope that the removal effects could persist if they succeeded in lowering crayfish densities over large stretches.

Our findings have important implications for the management of invasive crayfish populations to mitigate their ecological impacts. To the authors' knowledge, this study represents the first evidence that the removal of signal crayfish, at intensities which are easily feasible during control attempts or commercial crayfishing operations, may be beneficial for a range of sympatric aquatic macroinvertebrate biota. The encouraging trends from the flanking sections suggest that these benefits could potentially be maintained through regular reductions in crayfish density and extend beyond the limit of the trapped area. Based on the results of this study, it seems likely that noneradication crayfish control may benefit a wide range of taxa, supporting Simberloff's (2009) position that successful control of an invasive species, and restoration of ecosystem function, does not necessarily require that the invasive population be eradicated.
